# The effect of nutrition and health behavior change communication through community‐level actors on the nutritional status of pregnant women in the Ambo district, Ethiopia: A randomized controlled trial

**DOI:** 10.1002/fsn3.3643

**Published:** 2023-08-28

**Authors:** Mitsiwat Abebe Gebremichael, Tefera Belachew Lema

**Affiliations:** ^1^ Public Health Department, College of Medicine and Health Sciences Ambo University Ambo Ethiopia; ^2^ Human Nutrition Unit, Population and Family Health Department, College of Public Health and Medical Sciences Jimma University Jimma Ethiopia

**Keywords:** Ambo, Ethiopia, nutrition and health behavior change communication, nutritional status, pregnant women

## Abstract

Pregnant women in Ethiopia often had trouble understanding the normal nutrition and health information given by health professionals and found it inconsistent. Undernutrition is still a significant public health problem as a result. Hence, this trial aimed to assess the effect of nutrition and health behavior change communication using the community‐level actors on the nutritional status of pregnant women. Cluster‐randomized controlled community trial with baseline and endline measurements were used. Endpoint data from 744 pregnant women (372 intervention and 372 control groups) were gathered, respectively. In the intervention group, the community‐level actors delivered the behavior change communication main message based on intervention protocol. The control group got nutrition information during their ANC visits but did not receive the behavior change communication intervention. Binary generalized estimating equations regression analyses adjusted for baseline covariates were used to test effects of the intervention on nutritional status. Following the trial's implementation, the prevalence of undernutrition in the intervention arm fell by 10% from the baseline (23.7% vs. 13.7%) and was 13.7% lower than in the control arm (13.7% vs. 27.4%, *p* = <.001). Pregnant women in the intervention group showed significantly lower risk of undernutrition than the control group (ARR = 0.21; 95% CI 0.14–0.30). This study showed that engaging community actors to communicate about nutrition and health behavior change was successful in enhancing pregnant women's nutritional status. Therefore, nutrition and health behavior change communication through the community‐level actors is recommended to improve the nutritional status of pregnant women.

## Abstract 2

Pregnant women in Ethiopia often had trouble understanding the normal nutrition and health information given by health professionals and found it inconsistent. Undernutrition is still a significant public health problem as a result. Hence, this trial aimed to assess the effect of nutrition and health behavior change communication using the community‐level actors on the nutritional status of pregnant women.

## 1 INTRODUCTION

Nutrition has a significant impact on health throughout one's life. The nutritional and health state of the mother before conception and during pregnancy is critical in defining her own and her child's health and well‐being (Black et al., 2008). According to the essential nutrition actions (ENA) framework, pregnant women should get an optimal quantity and quality of nutrition, important micronutrient supplementation, disease prevention and treatment practices, and supportive lifestyle and care practices during pregnancy (McClure et al., 2013; MOG, 2011; WHO, 2013).

Undernutrition is a major public health concern for women of reproductive age and children, particularly pregnant women, because it has a negative impact on pregnancy outcomes (Dalky et al., 2018). Undernutrition in pregnant women can lead to a variety of problems. It makes women more vulnerable to a variety of disorders, including the chance of miscarriage, intrauterine growth retardation, low birth weight, and infant morbidity and mortality (Imdad & Bhutta, 2012). Despite this, maternal undernutrition is very common around the world, especially in Asian and sub‐Saharan African countries (Biswas et al., 2019). Undernutrition among pregnant women is prevalent in China (21%), Sri Lanka (15%), and Nigeria (10%–40%) (Adikari et al., 2016; Liu et al., 2015; Ugwa, 2016).

Ethiopia is one of the countries in sub‐Saharan Africa with the highest rates of maternal and child undernutrition. According to the 2016 Ethiopian demography and health survey (EDHS), 22.4% of women of reproductive age were undernourished (CSA & ICF, 2016). In Ethiopia, undernutrition among pregnant women ranges from 19.5% to 41.2%. It was reported in Dessie, Northern Ethiopia (19.5%), Silte Zone, Southern Ethiopia (21.8%), Ethiopia's Central Refit Valley (31.7%), and Shashemene, Southern Ethiopia (41.2%) (Belete & Firehiwot, 2016; Diddana, 2019; Mariyam & Dibaba, 2018; Muze et al., 2020).

This study used the community‐level actors (CLAs) to communicate behavior change as an implementer of the intervention. They are volunteers, especially support pregnant women, and in particular encourage them to give birth in health facilities where skilled birth attendants are available (FMOH, 2016). However, to the level of researcher's knowledge, no evidence was found on NHBCC interventions delivered through CLA whether to improve nutritional status of pregnant women.

Communication about nutrition and healthy behavior change is one of the most well‐known health promotion strategies (NHBCC). It is a collaborative strategy that entails working with individuals and groups to create communication channels that promote constructive behavior and create an environment where those behaviors may be adopted and sustained (Canavati et al., 2016; Middleton et al., 2013).

Numerous investigations have found that sociodemographic factors, such as age, wealth index, residence, size of farmland, household size, illiteracy or low education, occupation, and household food security, as well as maternal and health service‐related factors, such as years at marriage, ANC visits, parity, gravidity, gap duration between pregnancies, and dietary practices, are commonly factors affecting the nutritional status of pregnant women (FDRE & USAID, 2012; Guyon & Quinn, 2011; UNICEF, 2016; USAID & JSI, 2015; USAID/ENGINE & Save the Children, 2014). What our study makes different from other researchers was that it takes into account women's decision‐making power, knowledge, and attitude regarding nutrition and health based on essential nutrition action concepts.

There was a dearth of information on how NHBCC affected pregnant women's nutritional status in Ethiopia. To the best of our knowledge, no research has been done on how NHBCC is affecting pregnant women's nutritional status through community‐level actors. So, the objective of this research was to ascertain how NHBCC provided by community‐level actors affected pregnant women's nutritional status in the Ambo district. The study's findings may be used to inform policymakers and planners about the nutritional status of pregnant women at the national and regional levels.

## 2 METHODS AND MATERIALS

### 2.1 Study setting and period

**FIGURE 1:**
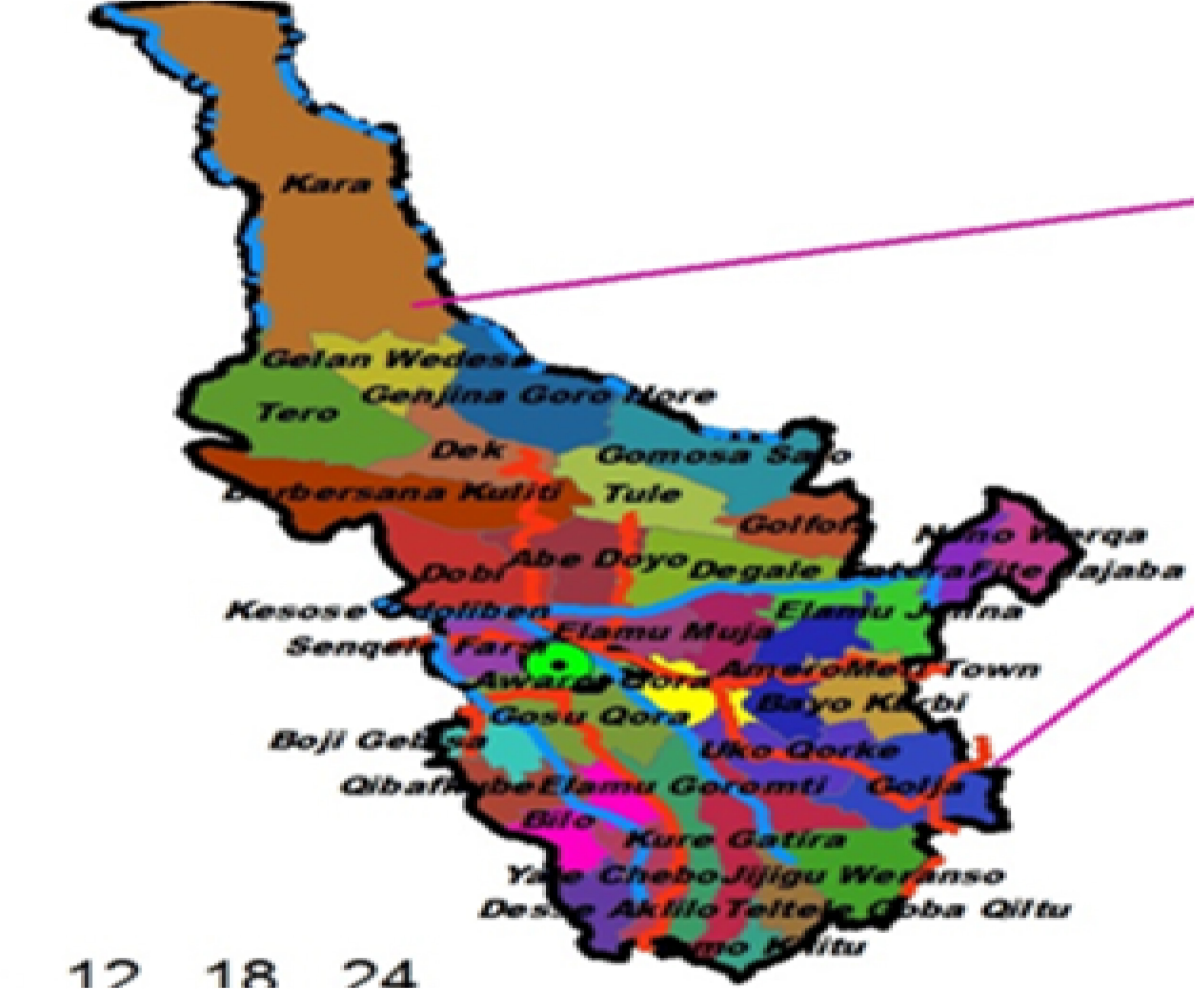
Map of study area, Ambo district with respective kebeles (*Source*: West Shoa Zone, 2017).

The study was conducted in Ambo district from June 2018 to December 2018. Ambo district is located in West Shoa Zone, Oromia Regional State, and west‐central Ethiopia. Ambo district is one of 22 districts in the west Shoa Zone, located at a latitude and longitude of 8°59′ N 37°51′ E, and divided into 39 kebeles (Ethiopia's smallest administrative units), 6 of which are urban and 33 of which are rural. Ambo town is the capital town of both the district and the zone, which is located 114 km from Addis Ababa, the capital city of Ethiopia. According to the CSA (2022) census report, the district has a total population of 152,143. Of this number, 5668 are men and the remaining 76,475 are women (CSA, 2022). Based on 2017 district health office data, it has 37,454 and 6976 reproductive age group and pregnant women, respectively (West Shoa Zone, 2017). In the district, there are two public hospitals, eight health centers, and six health posts. Livestock (like cattle, sheep, goats, and poultry) and cereals and pulses (like maize, wheat, teff, barley, and sorghum, beans, lentils, and peas) and vegetables and fruits (like cabbage, collard greens, tomatoes, potatoes, mangoes, and avocados) are the dominant agricultural livelihoods in the districts (ABAD, 2021) (Figure 1).

### 2.2 The context

In this study context, the CLAs is a group of organized women who represent the six nearest households (five members and one leader) in groups of five (1–5 networks) and in larger groups, usually 30 households, known as women's development groups (WDG). It was first introduced in Ethiopia in 2010. They are very supportive of pregnant women, encouraging them to give birth at health facilities with competent birth attendants (FMOH, 2016).

CLAs are volunteers who have the ability to improve access to primary health care (PHC) in Ethiopia and complement the work of health extension workers (HEWs)(JU, UG, & LSHTM, 2016) and there has been a considerable improvement in mother and child health and service usage since CLAs were introduced to the country; they are also more connected to the community and are seen as a role model by women (CSA, 2011; CSA & ICF, 2016). In the Oromia Region, a total of 195,864 CLA groups and 880,975 one‐to‐five networks were established in 2014 (FMOH, 2016).

The one‐to‐five network functions as a forum for the exchange of concerns, priorities, problems, and decisions related to the health status of women (Teklehaimanot & Teklehaimanot, 2013). On average, there are approximately 30 CLA team leaders and 200 CLA network leaders in each kebele (Summary & Bissau, 2013).

### 2.3 Study design

**FIGURE 2:**
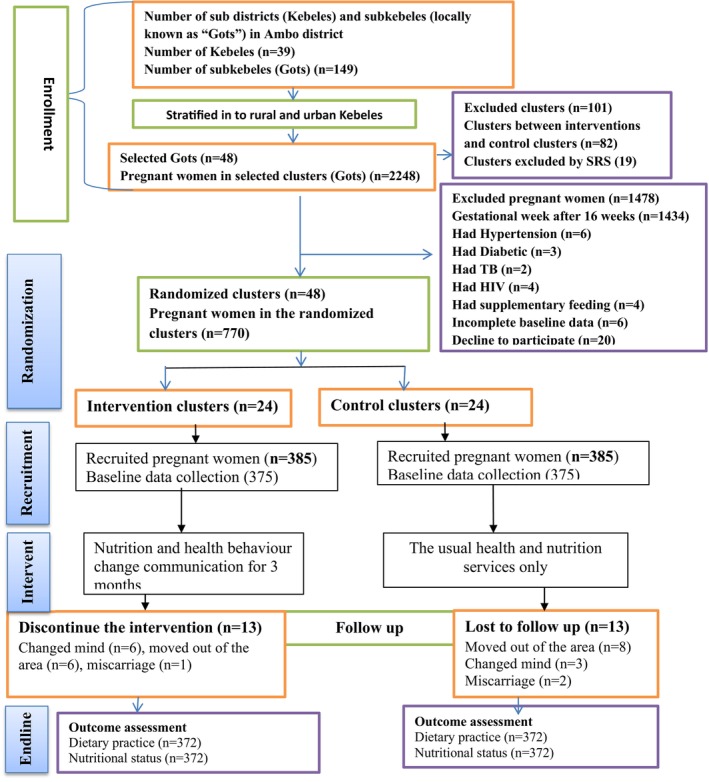
This diagram depicts the flow of study participants through the trial according to the criteria recommended in the CONSORT guideline.

A two‐arm parallel design cluster‐randomized controlled community trial was carried out. The intervention was delivered in community settings. Consolidated Standards of Reporting Trials (CONSORT) guideline was used for reporting the result (Campbell et al., 2012) (Figure 2).

### 2.4 Sample size determination

The sample size was calculated using the G Power 3.1.9.2 program based on the following assumptions. α error probability = 0.05; power = 0.8; an effect size of 0.5; allocation ratio (N1/N2) = 1; design effect of 2; 10% loss to follow‐up. According to a study done in Shashemene, West Arsi Zone, Ethiopia, the proportion of undernutrition (P1) 0.431 (Belete & Firehiwot, 2016) while prevalence P2 was 0.281, by assuming a 15% difference between p1 and P2 (West et al., 2011), the final sample size was 758 pregnant women (379 pregnant women in the intervention group and 379 pregnant women in the control group).

However, the sample size determined for the first outcome of this trial (optimal dietary practice) gave the largest sample sizes 770 (385 women in the intervention group and 385 women in the control group), which were included in the trial.

### 2.5 Sampling and randomization

The Ambo district's clusters (also known as sub‐kebeles or “gots”) served as the trial's randomization unit, and the pregnant women living in those clusters served as the observational units. The entire numbers of kebeles in the district were divided into rural and urban areas. From the 39 kebeles (6 urban and 33 rural) that were already present, 12 kebeles (2 urban and 10 rural) were chosen using simple random sampling (SRS). Each kebele has its own clusters (or “gots”), and a total of 149 different clusters are available in the 12 selected kebeles. We used a proportionate stratified sampling technique to obtain clusters from each kebele. First, the non‐adjacent clusters (gots) of each kebele were identified in the district. The clusters that were somewhat far from one another were then selected from all of the non‐adjacent clusters and included in the study. Based on that, 48 clusters were randomly selected from among all those that were available in the district. As a result, each arm of this investigation had 24 clusters. Hence, larger distance between clusters and non‐adjacency were the two selection criteria. Although it is difficult to depict the distance in figures between included clusters, we left at least one cluster between the intervention and control clusters that we employed for our research as a buffer zone to prevent information contamination. Furthermore, all eligible pregnant women in one cluster were enrolled in the same arm (either in the intervention or control arms). Finally, clusters were randomly assigned in a 1:1 ratio to the intervention and control arms. Actual and potential confounding factors were evenly distributed across the study arms due to the stratification and randomization techniques, which ultimately ensured the comparability of the arms. There were typically 12–17 pregnant women in each cluster.

### 2.6 Implementation and intervention allocation

Generation of the allocation sequence and the randomization of clusters was done by a statistician that was blinded to study groups and not participated in the research. Health extension workers in a particular district enrolled and assigned study participants to interventions.

### 2.7 Concealment

The cluster allocation was concealed from the data collectors by not disclosing it to them, excluding them from the trial implementers, and not residing in any of the clusters. During enrollment and the gathering of baseline data, trial participants were kept blind to the existence of an intervention. Data collectors, CLAs, and participants were all provided with no information about the study's hypothesis. Supervisors on the field were unaware of the outcome of the investigation. However, due to the design and nature of the intervention under study, blinding of the trial participants receiving the intervention and the CLA was not possible.

### 2.8 Recruitment

Through the house‐to‐house survey, pregnant women who were eligible were found by asking about the first day of their last menstrual cycle and utilizing a pregnancy test to confirm their pregnancy (a lab test). The trial included all pregnant women in the selected clusters who met the inclusion criteria. Women who were pregnant before 16 weeks of gestation and who had no plans to leave the study area before giving birth met the inclusion criteria. Pregnant women with chronic diseases such as hypertension, diabetes, TB, and HIV/AIDS, as well as those participating in supplemental feeding programs, were excluded from the study because these treatments would have an impact on nutritional status and bias the findings.

### 2.9 The intervention

Pregnant women in the intervention clusters received NHBCC for 3 months whereas pregnant women in the control groups (clusters) did not receive the NHBCC intervention but were received nutrition education during their ANC visits and any intervention at the community level by health extension workers though it may not have been as thorough as other care like diagnose and treat diseases or complications. This service was available to pregnant women in both the control and intervention groups. They underwent the same assessments and the same duration of observation as the intervention group. The following were the languages used throughout the intervention period: (Afan Oromo and Amharic, the local language). The intervention had three parts.

#### 2.9.1 Part 1: Training of the CLAs leaders

The researchers continued the intervention technique with the assistance of health extension workers to find CLAs. A total of 24 CLA leaders were recruited from the intervention clusters and centrally trained. The investigator developed a protocol for pregnant women based on the Essential Nutrition Action Framework and a framework for promoting maternal nutrition developed by the Manoff Group for developing countries. CLAs were trained using this protocol for pregnant women for 1 week (Chowdhury et al., 2017; MOG, 2011; WHO, 2013). The purpose of the training was to empower CLAs and support groups, with action‐oriented knowledge, attitudes, and behaviors to effectively communicate, support, and negotiate with pregnant women to adopt recommended practices. The training comprised both theoretical and practical demonstrations, and CLAs leaders adhered to the criteria (based on the protocol) to guarantee the acceptance of the intervention. During the group training of CLAs leaders, role‐playing, participatory meal preparation, and mock NHBCC sessions were utilized. The researchers developed a key assessment checklist including both theory and skill to lessen variation across CLAs leaders. The knowledge and skill of the community‐level actors leader were evaluated before and after training as well as through practical evaluations. To make sure that everyone was performing at a similar level after the training, the authors conducted standardization tests.

#### 2.9.2 Part 2: Group training of mothers by CLA leaders

The community's intervention clusters were the setting for the pregnant women's intervention. However, the pregnant women and the CLAs came to an understanding over the precise location of the NHBCC training (so that they have traveled to a common site but not out of the intervention clusters). The intervention lasted 3 months and was carried out for 1 to 2 hours once every 2 weeks on days off (religious holidays and weekends). In addition, the researchers monitored the intervention activities once every 2 weeks. Given that each cluster typically contained 12–17 pregnant women, one CLA was in charge of each cluster.

The nutrition and health practice message on consuming more food throughout pregnancy (increasing meal frequency and portion size with gestational age) and eating a variety of food from vegetable, fruit, and animal sources is included in the intervention's core content; Due to their education, the selected CLAs received prepared teaching materials, a schedule, and topics to cover during each contact (Table 1).

**TABLE 1:** Schedule and topics to be covered in each encounter, for pregnant women, Ambo district, Ethiopia, 2018.

Topics to be covered	Weeks	Age of trimester	Mode of BCC	Responsible person
Focused on basic nutrition, preparation, eating on a variety (diversified) of food from vegetable, fruit, and animal sources.	The first 2 week	First trimester of pregnancy	Theory	CLA
Utilization of iodized salt, decrease consumption of iron‐inhibiting foods, decreasing/avoiding of alcohol consumption, importance of taking iron/folate and on food taboos, reducing heavy workload	The second 2 week		Revision of the first 2 week and practical demonstration was applied	CLA and pregnant women
Sleeping under an insecticide‐treated bed net (ITN), seeking treatment from health institution if developed illness, personal and food hygiene, important of reducing heavy workload and taking day rest, self‐decision‐making on food and health, importance of obtaining support from the family/Community during pregnancy, utilization of health care services, and birth preparedness and complication readiness	The third 2 week The fourth 2 week	Second trimester of pregnancy	Theory Revision of the third 2 week and practical demonstration was applied	CLA CLA and pregnant women
The identified gaps will be covered	Fifth and sixth 2 weeks	Early third trimester of pregnancy	NHBCC was given based on the identified gaps	CLA and pregnant women

The consequences of disregarding the aforementioned message were also covered at each NHBCC session. CLAs leaders also evaluated participants' knowledge of and attitudes toward a healthy nutrition and lifestyle at each NHBCC session. A NHBCC letter was then distributed in accordance with the gaps that were found. A message about eating habits focused on easily accessible, socially acceptable, and reasonably priced meals. The CLAs leaders received posters and pamphlets with pertinent imagery to display to pregnant mothers in addition to the NHBCC messages.

Important messages were written in Afan Oromo and Amharic (local) and illustrated on leaflets distributed to pregnant mothers. If a woman could not read, it was advised that she have someone read the leaflet to her at home or in the neighborhood.

A practical learning demonstration included meal preparation, providing samples of iron and folate supplements, the kind and timing of adding iodized salt while cooking, how to use an insecticide‐treated bed net, and personal and food hygiene. To show how meals are prepared, participants were encouraged to share food from their personal kitchens. Pregnant women actively recognized the food types and preparation techniques they should avoid based on this visual display.

Six NHBCC sessions were attended by each pregnant woman in the intervention group over the course of her pregnancy. In a personal training diary, the CLAs leaders monitored and documented adherence to the NHBCC sessions (attendance sheet).

#### 2.9.3 Part 3: Home visits

Six home visits were done by each CLA leader in the intervention clusters that aimed to change the behavior of pregnant mothers and their families (every 2 weeks, for a duration of 2 days each). Every pregnant woman received individualized counseling and support during each home visit, which also served to reinforce the adoption of the practices she had been taught during the group training sessions, watch preparation practices, demonstrate cooking techniques, correct harmful practices, and provide suitable feedback centered on the protocol's key recommendations (Table 2).

**TABLE 2:** Nutrition and Health BCC key messages in the intervention clusters.

No.	Key messages	BCC techniques applied	Supportive intervention
1	*Quantity‐related messages*		Showed posters and brochures to group and gave leaflets with key messages to woman
1.1	Increase meal frequency (at least one extra diet)	NH education and demonstration	
1.2	Increase portion size with gestational age	Counseling and nutrition and health communication	
1.3	Avoidance/reducing of sharing of food with others	Counseling/Group discussions	
2	*Quality‐related messages*		
2.1	Consumption of fruits, vegetables, animal products	Counseling and NH communication	
2.2	Decrease consumption of iron‐inhibiting foods, such as tea (coffee) with meals.	Counseling/Group discussions	
3	*Micronutrient intake‐related messages*		Showed posters and brochures to group and gave leaflets with key messages to woman
3.1	Take daily supplements of iron and folic acid supplements during pregnancy for at least 3 months Exhibiting samples of iron/folate tablets	nutrition and health communication and demonstration	
3.2	Use iodized salt for the whole family with appropriate usage Exhibiting samples of iodized salt	NH education and demonstration	
4	*Disease prevention and treatment practice (high priority for malaria and worms) related messages*		
4.1	Pregnant women with fever need to be taken to a health facility for immediate treatment.	Group discussion/communication	
4.2	Keeping the environment clean	Counseling	
4.3	Wash hands with soap during key contact moments and drink treated water	NH education and demonstration	
4.4	Keeping food and food Containers Clean	NH Education and demonstration	
5	*Supportive lifestyle and care‐related messages*		
5.1	Had appropriate work load and rest during pregnancy	Group discussion	
5.2	Improve decision‐making power in food and her own health	Group discussion	
5.3	Get support from family or other individual during pregnancy	Group discussion/awareness	
5.4	Utilization of health care services (ANC follow‐up, Plan to deliver at health institution, and PNC follow‐up)	Group discussion/counseling	
5.5	Birth preparedness and complication readiness	Counseling/Group discussion	

Supervisors monitored and supervised the CLA leaders. The researcher was in charge of providing general guidance and monitoring. The entire study period's activities are displayed in (Table 3).

**TABLE 3:** Schedule of activities during the study period.

Activities	Time points in months										
	1	2	3	4	5	6	7	8	9	10	11
*Pre‐enrollment*											
Cluster selection	X										
Allocation	x^I+C^										
Eligibility screen	x^I+C^										
Informed consent		x^I+C^	x^I+C^								
*Enrollment*		x^I+C^	x^I+C^								
*Baseline data collection*		x^I+C^	x^I+C^								
Sociodemographic data Nutrition and health knowledge, and attitude, and dietary practice MUAC											
*Intervention*											
Training of CLA leaders on (NHBCC)				x^I^							
Group training of Pregnant women on (NHBCC)				x^I^	x^I^	x^I^	x^I^				
Home visits				x^I^	x^I^	x^I^	x^I^				
*Follow‐up*				x^I+C^	x^I+C^	x^I+C^	x^I+C^	x^I+C^	x^I+C^	x^I+C^	
Process evaluation				x^I^	x^I^	x^I^	x^I^	x^I^	x^I^	x^I^	
*Endline data collection: (Impact evaluation)*											x^I+C^
Dietary practices											
Nutritional status											
*Supervision*		x^I+C^	x^I+C^	x^I^	x^I^	x^I^	x^I^	x^I^	x^I^	x^I^	x^I^

*Note*: I, Intervention groups; C, Control groups; I + C, Activities both in intervention and control groups.

Between May and June 2018, study participant recruitment and baseline data collection took place. From July 2018 to September 2018, the intervention was provided for the intervention clusters following the baseline survey. The collection of the endline data took place between December 2018 and January 2019.

NB: Process evaluation was done to keep track of the intervention implementation procedure, assess the effectiveness of CLA leaders, ascertain whether the intervention activities were carried out as planned, and determine the extent to which the intervention reached the trial participants who were intended to receive it.

### 2.10 Ethical statements

The study was approved by Jimma University's Institutional Review Board (IRB), and it was carried out in conformity with all applicable local, regional, national, and international guideline. The study area's district and zonal administration and health offices gave their approval for the project. Participants in the study were fully informed of the purpose of the trial and their right to decline before providing their written informed consent for participation (signed or a fingerprint for those who could not read or write). All information gathered was kept private. The participant's right to leave the research at any time was respected. The display of the results will in no way include any information that might be used to determine a person's identity. Authors declare that they have no conflicting interests. This community trial was registered with the Pan African Clinical Trial Registry with registration number PACTR201805003366358.

### 2.11 Data collection tools and procedures

Eight diploma‐trained nurses collected the data, and four female laboratory technicians were recruited to do pregnancy tests. The information, which includes sociodemographic and economic characteristics, maternal characteristics, knowledge, and attitudes about nutrition and health, dietary practices among pregnant women, and nutritional status, was gathered using a pre‐tested semi‐structured interviewer‐administered questionnaire. Data on sociodemographic, economic, and maternal factors were gathered at the baseline. We collected data on dietary practices and nutritional status both before and after the intervention.

The questionnaire was developed to assess knowledge and attitudes on nutrition and health and was adapted from the WHO Essential Nutrition Action Framework and the Manoff Group's formative research on maternal nutrition promotion in developing countries (MOG, 2011; WHO, 2013).

Twenty‐six questions were used to assess pregnant women's knowledge of nutrition and health. Each participant received a knowledge score based on the number of questions throughout the knowledge evaluation phase that were correctly answered. A “1” was assigned to each correct response, while a “0” was assigned to each incorrect response. If a pregnant woman's score fell below the third tertile, her nutrition and health knowledge was considered to be poor, and if her score fell inside the third tertile, her nutrition and health knowledge was considered to be good (Belachew et al., 2013).

Twenty attitude questions were used to assess attitudes toward nutrition and health. Pregnant women who agreed to respond to questions about their opinions received a score of 2, a score of 1 for being neutral, and a score of 0 for disagreeing, according to the Likert scale. Each pregnant woman's overall attitude score was calculated by adding the answers to the 20 questions about attitudes. If a pregnant woman's score was below the third tertile, it was assumed that she had unfavorable attitudes; however, if it was inside the third tertile, it was assumed that she had favorable attitudes (Demilew et al., 2020b).

Using a questionnaire adapted from the Ethiopian Demographic and Health Survey (EDHS), the household wealth index and women's decision‐making power were assessed (EDHS, 2011). Principal components analysis (PCA) was used to construct the latent factors describing the wealth data, which were subsequently categorized into wealth trials. In assessing women's decision‐making powers, eight questions were used. Each question received a code of one when a decision was made by the woman individually or jointly with her husband; otherwise, each question received a code of zero. The ability of a woman to decide was classified using the mean (CSA & ICF, 2016).

The household food insecurity access scale (HHFIAS), which was validated in developing countries (Gebreyesus et al., 2015), is used to measure household food insecurity. The state of food security was assessed using nine previously validated questions that received a minimum score of 0 and a maximum score of 27. Depending on how many of the first 2, 2 to 10, 11 to 17, and >17 food insecurity indicators a household encountered, it was categorized as being food secure, somewhat, moderately, or severely food insecure.

The primary outcome variable (i.e., dietary practice) was assessed using a semi‐structured questionnaire that was adapted and modified from the FANTA (FANTA III, 2018) and other literature (Belachew et al., 2013; Daba et al., 2015; Gatahun, 2015; Workicho et al., 2016). The Dietary Diversity Score, Animal Source Food Consumption, and Increased Meals (both in frequency and amount) were used to assess dietary practices. When women increased in frequency and amount, had a higher DDS, and a high ASF consumption, their dietary practices were optimal; conversely, when they decreased in frequency and amount, had a lower DDS, or had a lower ASF consumption, their dietary practices were suboptimal (Belachew et al., 2013).

Nutritional status of the pregnant women was assessed by measuring the mid‐upper‐arm circumference (MUAC) as a secondary outcome of this study. MUAC is a better indicator of pre‐pregnancy body fat and the nutritional status of pregnant women than body mass index (Dadi & Desyibelew, 2013; Fakier et al., 2017). Therefore, in this study, MUAC was used to assess the nutritional status of pregnant women. Post‐intervention data were measured from 36 to 37 weeks of pregnancy. MUAC was measured using a non‐stretchable MUAC tape. The upper left arm's MUAC was taken with no clothing on it. The left arm was used as it shows malnutrition while the right arm, which is frequently used, will show lean muscle mass as a result of work (Fakier et al., 2017). During the procedure, the midpoint of the upper arm was located by flexing the women's elbows to 90° with the palm facing upwards. Then the distance from the acromion to the olecranon processes was measured and the midpoint was marked with ink. Finally, the measuring tape was placed snugly around the arm at the midpoint mark while the arm was hanging freely, palm facing toward the thigh. Two measurements were taken and read to the nearest 0.1 cm on the same day for each study subject. Women with MUACs greater than or equal to 23 cm were considered normally nourished, whereas those with MUACs less than 23 cm were considered undernourished (Ghosh et al., 2019; Tang et al., 2016).

Pregnant women (*n* = 770) provided baseline data from June 1–21, 2018, while pregnant women (*n* = 744) provided endpoint data in December 2018.

### 2.12 Data processing and analysis

Before entering data, data were manually checked for completeness and consistency during data collection. Then, it was entered into EPI data version 3.1 and exported to SPSS for Windows version 23 for cleaning and analysis. First, descriptive statistics like mean and Standard Deviation was done for continuous variable and frequency and percentage for categorical data. Multicollinearity was checked using variance inflation factors (VIF) and there was no Multicollinearity between independent variables.

The effect of intervention was measured at the endpoint of follow‐up. A chi‐square test was performed to compare the baseline characteristics of the intervention and control groups. The endline measurements were subtracted from baseline measurements for both intervention and control groups and the Comparisons of differences in differences of MUAC within the intervention and control groups were done using paired sample *t*‐tests and the comparisons of differences in differences of dietary practice and MUAC between the intervention and control groups were done using independent samples *t*‐tests. For the differences in proportions between intervention and control groups at endline chi‐square test was used. Binary GEE regression analyses were used to test effects of the intervention on MUAC of pregnant women. Mean differences and relative risks (RR) with 95% confidence interval (CI) were computed as a measure of intervention effects for continuous and categorical variables, respectively, on the nutritional status (undernutrition) of pregnant women. The adjusted effect measures were considered as the main result. The intention to treat analysis was performed in this study. The per‐protocol analysis includes all the study participants who adhered to the predetermined guideline. Therefore, in this study, women who attended six NHBCC sessions and gave endline data were included in the analysis.

### 2.13 Data quality control

Prior to the actual data collection, the questionnaire was pre‐tested among 5% of the sample who were not included in the final main sample. When the Cronbach's alpha value of the questionnaire was checked, it was found to be >0.7 (i.e., 0.81 for knowledge, 0.9 for attitude, and 0.76 for women's decision‐making powers), indicating that it is appropriate for use in the study. The objective of the study, the data collection method, the contents, and the measurements were covered in a 3‐day training session for data collectors and supervisors. To lower inter‐observer error, MUAC measurements undergo a standardization exercise. The four supervisors supervised the CLA's leaders every 2 weeks, and the investigator monitored all of their activities. Pregnant women in each cluster had the same number and frequency of NHBCC sessions, and contact times within each intervention group were similar. Field supervisors conducted a random assessment of 5% of the sample, informed the data collectors of a potential measurement issue, and corrected the issue on the spot. To better understand the questions, data in the local languages (Afan Oromo and Amharic) were gathered.

## 3 RESULTS

From the 770 pregnant women who enrolled in the trial, 744 (96.5%) were included in the analysis (362 in the intervention group and 372 in the control group). For the reasons listed below, 13 pregnant women in the intervention group discontinue participating in the intervention: Six women failed to attend to every NHBCC session; four decided not to take part in the intervention; two left the area; one miscarried. Similarly, 8 women moved out of the area, 3 changed their minds, and 2 miscarried, resulting in the loss of 13 pregnant women in the control group to follow‐up (Figure 2).

### 3.1 Baseline characteristics

At the beginning of the study, there was no significant difference between the intervention and control groups in any of the sociodemographic and obstetric characteristics, demonstrating balance across trial groups (*p* > .05) (Table 4).

**TABLE 4:** Sociodemographic characteristics of pregnant women in control and intervention groups at the beginning of the study, Ambo district, Ethiopia, 2018.

Variable	Category	Intervention group (*n*1 = 372)	Control group (*n*2 = 372)	*p*
Number of clusters		24	24	
Residence	Rural	296 (49.1)	307 (50.9)	.303
	Urban	76 (53.9)	65 (46.1)	
Religion	Protestant	153 (48.4)	163 (51.6)	.228
	Orthodox	169 (49.1)	175 (50.9)	
	Others	37 (44.0)	47 (56.0)	
Age of the respondent (Years)	Mean ± SD	27.3 ± 4.23	27.4 ± 4.48	.560
Respondents' Occupation	Employed	19 (47.5)	21 (52.5)	.868
	House wives/ Daily laborers	307 (50.2)	305 (49.8)	
	Merchants	23 (54.8)	19 (45.2)	
	Farmers	22 (44.0)	28 (56.0)	
Women educational status	No formal education	144 (50.7)	140 (49.3)	.555
	1–4 Grade	83 (46.9)	94 (53.1)	
	5–8 Grade	92 (50.5)	90 (49.5)	
	9–12 Grade	40 (54.1)	34 (45.9)	
	Diploma and higher	10 (37.0)	17 (63.0)	
Husband educational status	No formal education	112 (51.6)	105 (48.4)	.680
	1–4 Grade	72 (51.4)	68 (48.6)	
	5–8 Grade	95 (50.5)	93 (49.5)	
	9–12 Grade	66 (46.2)	77 (53.8)	
	Diploma and higher	24 (42.9)	32 (57.1)	
Household size	Mean ± SD	4.36 ± 1.58	4.55 ± 1.70	.150
Household wealth tertile	Low	110 (50.5)	108 (49.5)	.920
	Medium	159 (48.8)	167 (51.2)	
	High	100 (50.0)	100 (50.0)	
Estimated time to reach health institution	<30 min	77(50.0%)	77 (50.0%)	.980
	30–60 min	147 (49.8)	148 (50.2)	
	> 60 min	145 (49.2)	150 (50.8)	
Parity	Mean ± SD	2.22 ± 1.53	2.27 ± 1.60	.659
Gravidity	Mean ± SD	3.36 ± 1.72	3.35 ± 1.73	.876
Start of ANC	No	154 (49.4)	158 (50.6)	.912
	Yes	215 (49.8)	217 (50.2)	

### 3.2 Dietary practices

Before the implementation of the trial, there was no statistically significant difference in the overall dietary practices, dietary diversity score, animal source food consumption, and frequency of meals between the two groups (Table 5).

**TABLE 5:** Baseline dietary practices of pregnant women in Ambo district in Ethiopia, 2018.

Variables	Intervention (*n* = 372)	Control (*n* = 372)	*p*
	Frequency (%)	Frequency (%)	
*Dietary practice*			
Optimal	102 (27.4)	96 (25.8)	.552
Suboptimal	270 (72.6)	276 (74.2)	
*Dietary diversity score*			
High	48 (12.9)	32 (8.6)	.058
Low	324 (87.1)	340 (91.4)	
*Animal source food consumption*			
High	46 (12.4)	49 (13.2)	.742
Low	326 (87.6)	323 (86.8)	
*Meal frequency*			
Adequate	95 (25.5)	90 (24.2)	.913
Inadequate	277 (74.5)	282 (75.8)	

### 3.3 Effect of the intervention on the dietary practices of pregnant women

At the end of this trial, the proportion of pregnant women who had optimal dietary practices increased by 34.7% in the intervention group, a significant difference (*p* < .001). Even if the number of pregnant women who had optimal dietary practices slightly increased by 7.5% in the control group, it did not show a significant difference (*p* > .05). The average difference in optimal dietary practices between the two groups shows a significant difference; it was 27.2% (*p* < .001). Additionally, in the intervention group, DDS improved by 18.3%, whereas in the control group, it showed no improvement (−0.5% reduction), with an overall difference in 18.8% between the two groups (*p* < .001). In the same way, the proportion of women who took ASF increased by 19.7% in the intervention group while it decreased by 2.4% in the control group. The overall difference in ASF consumption was 22.1% between the two groups (*p* < .001). The proportion of women who took four or more meals increased by 43.0% in the intervention group, whereas it decreased by 6.7% in the control group. The overall difference in meal frequency was 49.7% between the two groups (*p* < .001) (Table 6).

**TABLE 6:** Differences between baseline and endline dietary practices and difference of the differences between intervention and control groups.

Variables	Intervention (*n* = 372)			Control (*n* = 372)			Difference of difference^b^
	Baseline	Endline	Difference (EL‐BL)^a^	Baseline	Endline	Difference (EL‐BL)^a^	
Overall optimal dietary practice	0.274	0.621	0.347	0.258	0.333	0.075	0.272**
High DDS	0.129	0.312	0.183	0.086	0.081	−0.005	0.188**
High ASF	0.124	0.321	0.197	0.132	0.108	−0.024	0.221**
Adequate meal frequency	0.255	0.685	0.430	0.242	0.175	−0.067	0.497**

Abbreviations: BL, baseline; EL, endline.

^a^ The difference was calculated by subtracting end‐line values from baseline values.

^b^ The difference of differences was calculated by subtracting difference in the controls from difference in the intervention groups.

** *p* < .001.

### 3.4 Nutritional status of pregnant women

#### 3.4.1 Effect of the intervention on the nutritional status of pregnant women

At the baseline, there was no statistically significant difference in the mean MUAC (23.9 ± 1.6 vs. 23.8 ± 1.7, *p* = .62) between the study groups. After the implementation of the trial, the mean MUAC in the intervention arm has increased by 40% from the baseline (23.9 ± 1.6 vs. 24.3 ± 1.7, *p* < .001). *t*‐Test results showed that the intervention improved the mean MUAC by 80% (Table 7).

**TABLE 7:** Differences between baseline and endline measurements of MUAC and difference of the differences between the intervention and control groups.

Variable	Intervention (*n*1 = 372)			Control (*n*2 = 372)			Difference of difference^b^	*p* *
	Baseline	Endline	Difference (EL‐BL)^a^	Baseline	Endline	Difference (EL‐BL)^a^		
	Mean ± SD	Mean ± SD	Mean ± SD	Mean ± SD	Mean ± SD	Mean ± SD	Mean ±SE	
MUAC	23.9 ± 1.6	24.3 ± 1.7	0.4 ± 1.3	23.8 ± 1.7	23.4 ± 1.9	−0.4 ± 1.4	0.8 ± 0.6	<.001*

Abbreviations: BL, baseline; EL, Endline; MUAC, mid‐upper‐arm circumference; SD, standard deviation; SE, standard error.

^a^ The difference was calculated by subtracting end‐line values from baseline values.

^b^ The difference of differences was calculated by subtracting difference in the controls from difference in the intervention groups.

* *p* Values were generated from Paired *t*‐test statistics.

At the baseline, there was no statistically significant difference in the prevalence of undernutrition (23.7% vs. 24.0%, *p* = .93) between the two groups. After the implementation of the trial, the prevalence of undernutrition was 13.7% lower in the intervention group compared with the control arm (13.7% vs. 27.4%, *p* = <.001). The intra‐cluster correlation (ICC) coefficient was closer to zero (.018). This revealed that individual‐level variables explained 98.2% of the nutritional status (Table 8).

**TABLE 8:** Nutritional status of pregnant women in the Ambo district, Oromia, Ethiopia, 2018.

Variables	Category	Intervention (*n*1 = 372)	Control (*n*2 = 372)	*p*
		*N* (%)	*N* (%)	
MUAC baseline	<23 cm (Undernutrition)	88 (23.7)	89 (24.0)	.932
	≥23 cm (Normal)	284 (76.3)	283 (76.0)	
MUAC endline	<23 cm (Undernutrition)	51 (13.7)	102 (27.4)	<.001
	≥23 cm (Normal)	320 (86.3)	270 (72.6)	
ICC	0.018			

Abbreviations: ICC, intra‐cluster correlation; MUAC, mid‐upper‐arm circumference.

The rates of undernutrition (13.7% vs. 27.4%; ARR = 0.21; 95% CI 0.14–0.30) were significantly lower in the intervention group as compared to the control groups (Table 9).

**TABLE 9:** Generalized estimated equation regression analyses on the pregnant women undernutritional status.

Variable	Study groups	Number (%)	ARR (95% CI)
MUAC	Control group	102 (27.4)	1
	Intervention group	51 (13.7)	0.21 (0.14–0.30)

*Note*: The model was adjusted for maternal (residence, age, occupation, education, wealth index, food security, number of ANC, estimated time to reach the nearest health institution, parity, and gravidity, baseline knowledge and attitude on nutrition and health), dietary practices, and household size.

Abbreviations: ARR, Adjusted relative risk; CI, confidence interval.

## 4 DISCUSSION

In this study, the community‐level actor's effect on pregnant women's nutritional status in Ethiopia's Ambo District, West Shewa Zone, was studied with respect to nutrition and health behavior change communication. Pregnant women's sociodemographic, obstetric, and dietary practices were similar at the beginning of the study. After the implementation of the trial, the mean MUAC in the intervention group has increased by 40% from the baseline, and the intervention improves the mean MUAC by 80%. This suggests that nutrition and health behavior change communication intervention via the community‐level actors improve nutritional status of pregnant women. This was supported by behavior change communication effort in the Lao PDR is yielding good maternal health outcomes by changing dietary practices with low nutritional indicators (The World Bank (WB), 2022). Similarly, this is in line with the findings of Sharifirad et al. (2013), who found that nutrition interventions had a positive effect on improving nutritional status during pregnancy when compared to traditional training.

Following the trial's implementation, the prevalence of undernutrition in the intervention arm fell by 10% from the baseline and was 13.7% lower than in the control arm. This is consistent with previous study that reported positive effects of nutritional counseling as an intervention strategy in improving the nutritional status during pregnancy (Garg & Kashyap, 2006). Similarly, the study done in West Gojjam Zone, Ethiopia, among pregnant women, even if it used a different strategy (i.e., follow guided counseling using the health belief model and theory of planned behavior constructs), revealed an improvement in the nutritional status of pregnant women (Demilew et al., 2020a).

The study conducted in Pakistan, even though it did not study a similar population, used a similar delivery strategy (i.e., the intervention was delivered by trained community health workers) and a similar study design (i.e., a cluster‐randomized controlled trial) and reported that maternal educational messages regarding appropriate complementary feeding improved the nutritional status of their infants after 30 weeks of educational interventions (Saleem et al., 2014). Similarly, according to USAID (2014) and Hornik et al. (2015), nutrition and health behavior change communication interventions can reduce undernutrition by influencing the adoption of nutrition‐specific behaviors (Hornik et al., 2015; USAID, 2014). This implies that the study's findings have practical relevance for preventing undernutrition among pregnant women.

When compared to the baseline prevalence, undernutrition was more common among pregnant women in the control group at the end of the study. Pregnant women in the control group experienced undernutrition to a greater extent than the baseline prevalence, according to other studies in Ethiopia (Kedir et al., 2016; Kumera et al., 2018), this is because after the first trimester of pregnancy, undernutrition affects a sizable portion of pregnant women, and the severity of undernutrition worsens with increasing gestational age.

The results of this study showed that pregnant women's nutritional status could be improved through nutrition and health behavior change communication with community‐level actors. Contrary to this intervention, nutrition and health counseling provided by healthcare professionals in healthcare facilities and by health extension workers in the community as ineffective in enhancing the nutritional status of pregnant women.

The variation in intervention strategies is what caused this discrepancy. This intervention included trimester‐based, facilitated group NHBCC training and then was followed by a home visit to observe their dietary behavior and identify the gap since it used intervention packages with core messages developed from the WHO ENA and the Mannoff Group frameworks for developing nations. According to our findings, the proportion of women in the intervention group who had optimal dietary practices was significantly higher than that of women in the control group. This suggests that our intervention helps pregnant women's dietary practices, which in turn can lead to an improvement in nutritional status.

Unfortunately, the education (or counseling) given by the healthcare staff in the healthcare facility and the community‐based health extension workers does not use core messages, is not trimester‐based, and is not followed by home visits to observe the participants' actual dietary behaviors. Consequently, in order to promote a positive behavior change, these findings point to the necessity of modifying how nutrition education and/or counseling is provided during pregnancy.

### 4.1 Strengths and limitations of the study

Some of this study's strengths include the use of a randomized controlled design with a large sample size, a combination of facilitated group training and home visits for each pregnant woman, and trimester‐based NHBCC messages. The intervention is less expensive because it was carried out by community‐level actors, and it is also more likely to be sustained because it took place in a setting where the community was supportive. This study does have some limitations, though. First, a double‐blind trial could not be done because of the nature of the study. Despite their lack of knowledge about its specific purpose, trial participants were aware that nutrition and health training were available in their community. Second, the intervention was rather short, and the post‐intervention result might not have lasted longer because our study only included two data collection sessions (at baseline and endline). In order to better understand the long‐term effects of the intervention, we recommend adding more follow‐up visits, measurements, and a longer intervention period to similar studies in the future.

## 5 CONCLUSIONS

This study revealed that nutrition and health behavior change using community‐level actors was effective in improving the nutritional status of pregnant women. Nutrition and health behavior change communication using community‐level actors is a low‐cost and suitable intervention to improve the nutritional status of pregnant women. The result also suggests that a well‐designed nutrition and health behavior change communication intervention incorporating core content on diet and health during pregnancy can reduce the prevalence of undernutrition.

## AUTHOR CONTRIBUTIONS

**Mitsiwat Abebe Gebremichael:** Conceptualization (lead); data curation (lead); formal analysis (lead); investigation (lead); methodology (lead); project administration (equal); resources (equal); supervision (lead); validation (lead); visualization (lead); writing – original draft (lead); writing – review and editing (lead). **Tefera Belachew Lema:** Conceptualization (supporting); data curation (supporting); formal analysis (supporting); methodology (equal); resources (supporting); supervision (supporting); validation (equal); visualization (equal); writing – original draft (supporting); writing – review and editing (supporting).

## FUNDING INFORMATION

Jimma University supported the authors to prepare posters and brochures with relevant images to display to pregnant women; leaflets with key messages written in (local) languages and illustrations; and transportation of researchers, data collectors, and perdium for health development armies during training and delivering the BCC messages to pregnant women.

## Data Availability

Raw SPSS Data were supplemented upon request of the corresponding authors.

## References

[fsn33643-bib-0001] ABAD . (2021). Agricultural bureau of Ambo District. Communication office.

[fsn33643-bib-0002] Adikari, A. , Sivakanesan, R. , Wijesinghe, D. , & Liyanage, C. (2016). Assessment of nutritional status of pregnant women in a rural area in Sri Lanka .

[fsn33643-bib-0003] Belachew, T. , Lindstrom, D. , Gebremariam, A. , Hogan, D. , Lachat, C. , Huybregts, L. , & Kolsteren, P. (2013). Food insecurity, food based coping strategies and suboptimal dietary practices of adolescents in Jimma zone Southwest Ethiopia. PLoS One, 8(3), e57643. 10.51371/journal.pone.0057643 23554864 PMC3595236

[fsn33643-bib-0004] Belete, Y. N. B. , & Firehiwot, M. (2016). Undernutrition and associated factors among adolescent pregnant women in Shashemene, west Arsi zone, Ethiopia: A community‐based study. Journal of Nutrition & Food Sciences, 6(1), 1‐7.

[fsn33643-bib-0005] Biswas, T. , Townsend, N. , Magalhaes, R. S. , Islam, M. S. , Hasan, M. M. , & Mamun, A. (2019). Current Progress and future directions in the double burden of malnutrition among women in south and southeast Asian countries. Current Developments in Nutrition, 3, 26. 10.1093/cdn/nzz026 PMC6584112 31240272

[fsn33643-bib-0006] Black, R. E. , Allen, L. H. , Bhutta, Z. A. , & Maternal and Child Under Nutrition Study Group . (2008). Maternal and child under nutrition: Global and regional exposures and health consequences. Lancet, 371, 243–260. 10.1016/S0140-6736(07)61690-0 18207566

[fsn33643-bib-0007] Campbell, M. K. , Piaggio, G. , Elbourne, D. R. , & Altman, D. G. (2012). Consort 2010 statement: Extension to cluster randomised trials. BMJ, 345(7881), 1–21. 10.1136/bmj.e5661 22951546

[fsn33643-bib-0008] Canavati, S. E. , de Beyl, C. Z. , Ly, P. , Shafique, M. , Boukheng, T. , Rang, C. , Whittaker, M. A. , Roca‐Feltrer, A. , & Sintasath, D. (2016). Evaluation of intensified behaviour change communication strategies in an artemisinin resistance setting. Malaria Journal, 15, 249. 10.1186/s12936-016-1276-8 27129496 PMC4851777

[fsn33643-bib-0009] Chowdhury, M. , Raynes‐Greenow, C. , Alam, A. , & Dibley, M. J. (2017). Making a balanced plate for pregnant women to improve birthweight of infants: A study protocol for a cluster randomised controlled trial in rural Bangladesh. BMJ Open, 7, e015393. 10.1136/bmjopen-2016-015393 PMC5724074 28827238

[fsn33643-bib-0010] CSA . (2011). Ethiopia demogeraphic and health survey. Central Statistical Agency (CSA), ORC Macro.

[fsn33643-bib-0011] CSA . (2022). Population size by sex, area and density by region, zone and Wereda. CSA.

[fsn33643-bib-0012] CSA & ICF . (2016). Ethiopia demographic and health survey. Central Statistical Agency (CSA) and ICF.

[fsn33643-bib-0022] Daba, G. , Beyene, F. , Garoma, F. , & Fekadu, H. (2015). Assessment of nutritional practice of pregnant mothers on maternal nutrition and associated factors in Guto Gida Woreda, east Wollega Zone, Ethiopia. JSTAR Journal, 2(3), 105–113.

[fsn33643-bib-0013] Dadi, A. F. , & Desyibelew, H. D. (2013). Undernutrition and its associated factors among pregnant mothers in Gondar town, Northwest Ethiopia. PLoS One, 14, e0215305. 10.1371/journal.pone.0215305 PMC6476509 31009475

[fsn33643-bib-0014] Dalky, H. , Qandil, A. , & Alqawasmi, A. (2018). Factors associated with undernutrition among pregnant and lactating Syrian refugee women in Jordan. Global Journal of Health Science, 10(4), 1–58.

[fsn33643-bib-0015] Demilew, Y. M. , Alene, G. D. , & Belachew, T. (2020a). Effect of guided counseling on nutritional status of pregnant women in West Gojjam zone, Ethiopia: A cluster‐randomized controlled trial. Nutrition Journal, 19(1), 38. 10.1186/s12937-020-00536-w 32345286 PMC7189500

[fsn33643-bib-0016] Demilew, Y. M. , Alene, G. D. , & Belachew, T. (2020b). Effects of guided counseling during pregnancy on birth weight of newborns in West Gojjam Zone, Ethiopia: A cluster‐randomized controlled trial. BMC Pediatrics, 20, 466. 10.1186/s12887-020-02363-8 33023521 PMC7542400

[fsn33643-bib-0017] Diddana, T. Z. (2019). Factors associated with dietary practice and nutritional status of pregnant women in Dessie town, northeastern Ethiopia: A communitybased cross‐sectional study. BMC Pregnancy and Childbirth, 19(1), 1–10. 10.1186/s12884-019-2649-0 31870426 PMC6929309

[fsn33643-bib-0018] EDHS . (2011). Ethiopia demographic and health survey 2011 (pp. 13). Central Statistical Agency and ORC Macro.

[fsn33643-bib-0019] Fakier, A. , Petro, G. , & Fawcus, S. (2017). Mid‐upper arm circumference: A surrogate for body mass index in pregnant women. South African Medical Journal, 107, 606–610. 10.7196/SAMJ.2017.vi7197.12255 29025451

[fsn33643-bib-0020] Fanta III . (2018). Food and Nutrition Technical Assistance. *Participant‐based survey sampling guide for feed the future annual monitoring indicators* .

[fsn33643-bib-0021] FDRE & USAID . (2012). Federal Democratic Republic of Ethiopia: A tool to support nutrition advocacy in Ethiopia: Ethiopia PROFILES estimates . (pp. 15–25). Final report.

[fsn33643-bib-0023] FMOH . (2016). *The health development army: Its origins*, *development and current status*. The Health Documentation Initiative.

[fsn33643-bib-0024] Garg, A. , & Kashyap, S. (2006). Effect of counseling on nutritional status during pregnancy. Indian Journal of Pediatrics, 73, 687–692. 10.1007/BF02898446 16936363

[fsn33643-bib-0025] Gatahun, E. A. (2015). Dietary diversity feeding practice and determinants among children aged 6‐23 months in Kemba Woreda, Southern Ethiopia implication for public health intervention. Journal of Nutrition & Food Sciences, 13, 13003. 10.1186/s40795-017-0202-y

[fsn33643-bib-0026] Gebreyesus, S. H. , Lunde, T. , Mariam, D. H. , Woldehanna, T. , & Lindtjørn, B. (2015). Is the adapted Household Food Insecurity Access Scale (HFIAS) developed internationally to measure food insecurity valid in urban and rural households of Ethiopia? BMC Nutrition, 1(2), 1–10.

[fsn33643-bib-0027] Ghosh, S. , Spielman, K. , Kershaw, M. , Ayele, K. , Kidane, Y. , Zillmer, K. , Wentworth, L. , Pokharel, A. , Griffiths, J. K. , Belachew, T. , & Kennedy, E. (2019). Nutritionspecific and nutrition‐sensitive factors associated with mid‐upper arm circumference as a measure of nutritional status in pregnant Ethiopian women: Implications for programming in the first 1000 days. PLoS One, 14, e0214358. 10.1371/journal.pone.0214358 30913234 PMC6435172

[fsn33643-bib-0028] Guyon, A. B. , & Quinn, V. J. (2011). Essential nutrition action frame work. Training guide for health workers. Core Group.

[fsn33643-bib-0029] Hornik, R. , Naugle, D. , Smith, W. , & Trevors, T. (2015). Investing in communication for nutrition related to agriculture in India . http://repository.upenn.edu/asc_papers/422

[fsn33643-bib-0030] Imdad, A. , & Bhutta, Z. A. (2012). Maternal nutrition and birth outcomes: Effect of balanced protein‐energy supplementation. Pediatric Perinatal Epidemiology, 26, 178–190. 10.1111/j.1365-3016.2012.01308.x 22742610

[fsn33643-bib-0031] JU, UG, & LSHTM . (2016). Facilitating accessible community‐oriented health systems: The Health Development Army in Ethiopia. Jimma University (JU), University of Glasgow (UG), London School of hygiene and tropical Medicine (LSHTM).

[fsn33643-bib-0032] Kedir, H. , Yemane, B. , & Alemayehu, W. (2016). Magnitude and determinant of malnutrtion among pregnant women in Eastern Ethiopia. Evidence from rural, community based setting. Maternal & Child Nutrition, 12, 51–63. 10.1111/mcn.12136 24985910 PMC6860056

[fsn33643-bib-0033] Kumera, G. , Gedle, D. , Alebel, A. , Feyera, F. , & Eshetie, S. (2018). Undernutrition and its association with socio‐demographic, anemia and intestinal parasitic infection among pregnant women attending antenatal care at the University of Gondar Hospital, Northwest Ethiopia. Maternal Health, Neonatology and Perinatology, 4, 1–10. 10.1186/s40748-18-0087-z 30214818 PMC6134711

[fsn33643-bib-0034] Liu, F. L. Z. Y. , Parés, G. V. , Reidy, K. C. , Zhao, W. Z. , Zhao, A. , & Chen, C. (2015). Nutrient intakes of pregnant women and their associated factors in eight cities of China: A cross‐sectional study. Chinese Medical Journal, 128(13). 1778‐1786. 10.4103/0366-6999.159354 PMC4733713 26112720

[fsn33643-bib-0035] Mariyam, A. F. , & Dibaba, B. (2018). Epidemiology of malnutrition among pregnant women and associated factors in central refit valley of Ethiopia, 2016. Journal of Nutritional Disorder, 8(1), 1–8. 10.4172/2161-0509.1000222

[fsn33643-bib-0036] McClure, E. M. , Goldenberg, R. L. , Dent, A. E. , & Meshnick, S. R. (2013). A systematic review of the impact of prevention of malaria in pregnancy on low birth weight and maternal anemia. International Journal of Gynecology & Obstetrics, 121, 103–109. 10.1016/j.ijgo.2012.1012.1014 23490427

[fsn33643-bib-0037] Middleton, P. F. , Lassi, Z. S. , Son Tran, T. , Bhutta, Z. , Bubner, T. K. , Flenady, V. , & Caroline, C. (2013). Nutrition interventions and programs for reducing mortality and morbidity in pregnant and lactating women and women of reproductive age: A systematic review Australian Research Centre for Health of Women and Babies (ARCH) (pp. 20–30). Robinson Institute, The University of Adelaide.

[fsn33643-bib-0038] MOG . (2011). *Guidance for formative research on maternal nutrition: Prepared for the infant and young child nutrition project* (pp. 4–6).

[fsn33643-bib-0039] Muze, M. Y. M. , Kedir, S. , & Mustafa, A. (2020). Prevalence and associated factors of undernutrition among pregnant women visiting ANC clinics in Silte zone, Southern Ethiopia. BMC Pregnancy Childbirth, 20(1), 1–8. 10.1186/s12884-020-03404-x PMC7678074 33213406

[fsn33643-bib-0040] Saleem, A. F. , Mahmud, S. , Baig‐Ansari, N. , & Zaidi, A. K. (2014). Impact of maternal education about complementary feeding on their infants' nutritional outcomes in low‐ and middle‐income households: A community‐based randomized interventional study in Karachi, Pakistan. Journal of Health, Population, and Nutrition, 32(4), 623–633. 25895196 PMC4438693

[fsn33643-bib-0041] Sharifirad, G. R. , Tol, A. , Mohebi, S. , Matlabi, M. , Shahnazi, H. , & Shahsiah, M. (2013). The effectiveness of nutrition education program based on health belief model compared with traditional training. Journal of Education and Health Promotion, 2, 15. 24083265 10.4103/2277-9531.112684 PMC3778581

[fsn33643-bib-0042] Summary, E. , & Bissau, G. (2013). *Ethiopia UNICEF annual report 2013‐Ethiopia* (pp. 1–62). https://www.unicef.orp/about/report/files/Ethiopia_COAR_2013.pdf

[fsn33643-bib-0043] Tang, A. M. , Chung, M. , Dong, K. , Terrin, N. , Edmonds, A. , Assefa, N. , Chetty, T. , Ramlal, R. , Christian, P. , West, K. , Janjua, N. , Wanke, C. , Deitchler, M. , & Maalouf‐Manasseh, Z. (2016). Determining a global mid‐upper arm circumference cutoff to assess malnutrition in pregnant women. FHI 360/Food and Nutrition Technical Assistance III Project (FANTA).

[fsn33643-bib-0044] Teklehaimanot, H. D. , & Teklehaimanot, A. (2013). Human resource development for a community‐based health extension program: A case study from Ethiopia. Human Resources for Health, 11(1), 39. 10.1186/1478-4491-11-39 23961920 PMC3751859

[fsn33643-bib-0045] The World Bank (WB) . (2022). Communicating behavior change for better nutrition in northern Lao PDR . https://www.worldbank.org/en/news/feature/2022/05/03/communicating‐behavior‐change‐for‐better‐nutrition‐in‐northern‐lao‐pdr

[fsn33643-bib-0046] Ugwa, E. A. (2016). Nutritional practices and taboos among pregnant women attending antenatal care at the general hospital in Kano, Northwest Nigeria. Annals of Medical and Health Sciences Research, 6(2), 109–114. 10.4103/2141-9248.181846 27213094 PMC4866363

[fsn33643-bib-0047] UNICEF . (2016). UNICEF conceptual framework of undernutrition . http://www.unicef.org/nutrition/training/2.5/4.html

[fsn33643-bib-0048] USAID . (2014). Evidence of effective approaches to social and behavior change communication for preventing and reducing stunting and anemia. Findings from a systematic literature review . https://www.spring‐nutrition.org/sites/default/files/publications/series/spring_sbcc_lit_review.pdf

[fsn33643-bib-0049] USAID & JSI . (2015). *Understanding the essential nutrition actions and essential hygiene actions framework* (pp. 1–5).

[fsn33643-bib-0050] USAID/ENGINE, & Save the Children . (2014). *Maternal diet and nutrition practices and their determinants engine: A project supported by the Feed the Future and Global Health Initiatives A report on formative research findings and recommendations for social and behavior change communication programming in the Amhara*, *Oromia*, *SNNP and Tigray regions of Ethiopia* (pp. 5–10).

[fsn33643-bib-0051] West, K. P., Jr. , Christian, P. , Labrique, A. B. , Rashid, M. , Shamim, A. A. , Klemm, R. D. W. , Massie, A. B. , Mehra, S. , Schulze, K. J. , Ali, H. , Ullah, B. , Wu, L. S. , Katz, J. , Banu, H. , & Akhter, H. H. (2011). Effects of vitamin a or beta carotene supplementation on pregnancy‐related mortality and infant mortality in rural Bangladesh. A cluster randomized trial. JAMA, 305(19), 1986–1995. 10.1001/jama.2011.656 21586714

[fsn33643-bib-0052] West Shoa Zone . (2017). Health Office.

[fsn33643-bib-0053] WHO . (2013). *Essential nutrition actions: Improving maternal*, *newborn*, *infant and young child health and nutrition* (pp. 3*–*45). https://apps.who.int/iris/handle/10665/84409 25473713

[fsn33643-bib-0054] Workicho, A. , Belachew, T. , Feyissa, G. T. , Wondafrash, B. , Lachat, C. , Verstraeten, R. , & Kolsteren, P. (2016). Household dietary diversity and animal source food consumption in Ethiopia: Evidence from the 2011 welfare monitoring survey. BMC Public Health, 16, 1192. 10.1186/s12889-016-3861-8 27884138 PMC5123272

