# Correction to “The effect of nutrition and health behavior change communication through community‐level actors on the nutritional status of pregnant women in the Ambo district, Ethiopia: A randomized controlled trial”

**DOI:** 10.1002/fsn3.3895

**Published:** 2023-12-07

Gebremichael, M. A., & Belachew Lema, T. (2023). The effect of nutrition and health behavior change communication through community‐level actors on the nutritional status of pregnant women in the Ambo district, Ethiopia: A randomized controlled trial. *Food Science & Nutrition*, 11, 7172–7187. https://doi.org/10.1002/fsn3.3643

In the originally published version of the article, an affiliation for Mitsiwat Abebe Gebremichae was missing: Department of Nutrition and Dietetics, Faculty of Public Health, Jimma University, Jimma, Ethiopia. The correct authors and affiliations appear below.

We apologize for this error.

Mitsiwat Abebe Gebremichael^1,2^, Tefera Belachew Lema^1^

^1^ Department of Nutrition and Dietetics, Faculty of Public Health, Jimma University, Jimma, Ethiopia

^2^Department of Public Health, College of Medicine and Health Sciences, Ambo University, Ambo, Ethiopia

